# D-2-hydroxyglutarate suppresses allergic sensitization in a murine model of experimental asthma

**DOI:** 10.1111/all.15809

**Published:** 2023-07-05

**Authors:** Anuj Tharakan, Ankit Kumar, Jeremy Allegood, Lauren Ashley Cowart, Daniel H. Conrad, Rebecca K. Martin

**Affiliations:** 1 Department of Microbiology and Immunology, Virginia Commonwealth University, Richmond, Virginia, USA; 2 Department of Biochemistry and Molecular Biology, Virginia Commonwealth University, Richmond, Virginia, USA; 3 Hunter Holmes McGuire Veteran’s Affairs Medical Center, Richmond, Virginia, USA

## To the Editor,

Asthma is a chronic inflammatory disease affecting the airways which is characterized by reversible airflow obstruction, bronchial hyperresponsiveness, and airway remodeling. In allergic asthma, this airway pathology is mediated by Type 2 cytokines (IL-4, IL-5, and IL-13) and allergen-specific IgE. IL-4, IL-5, and IL-13 are predominantly produced by lung resident T helper 2 (Th2) cells and Type 2 innate lymphoid cells. The production of allergen-specific IgE from B cells is driven by a subset of IL-4 and IL-13 producing T follicular helper cells termed T follicular helper 13 (Tfh13) cells.^1,2^ The initiation of these T-cell responses is mediated by dendritic cells (DCs). The mechanisms by which DCs regulate Th2 and Tfh13 polarization, however, are unclear. Several genome-wide association studies have identified that single-nucleotide polymorphisms (SNPs) in the *D2HGDH* locus are associated with allergic asthma and allergic rhinitis risk, with the G allele of rs34290285 associated with a lower risk of disease.^3^ The *D2HGDH* gene encodes the enzyme D-2-hydroxyglutarate dehydrogenase (D2HGDH), which converts D-2-hydroxyglutarate (D2HG) to α-ketoglutarate (α-KG).

**FIGURE 1:**
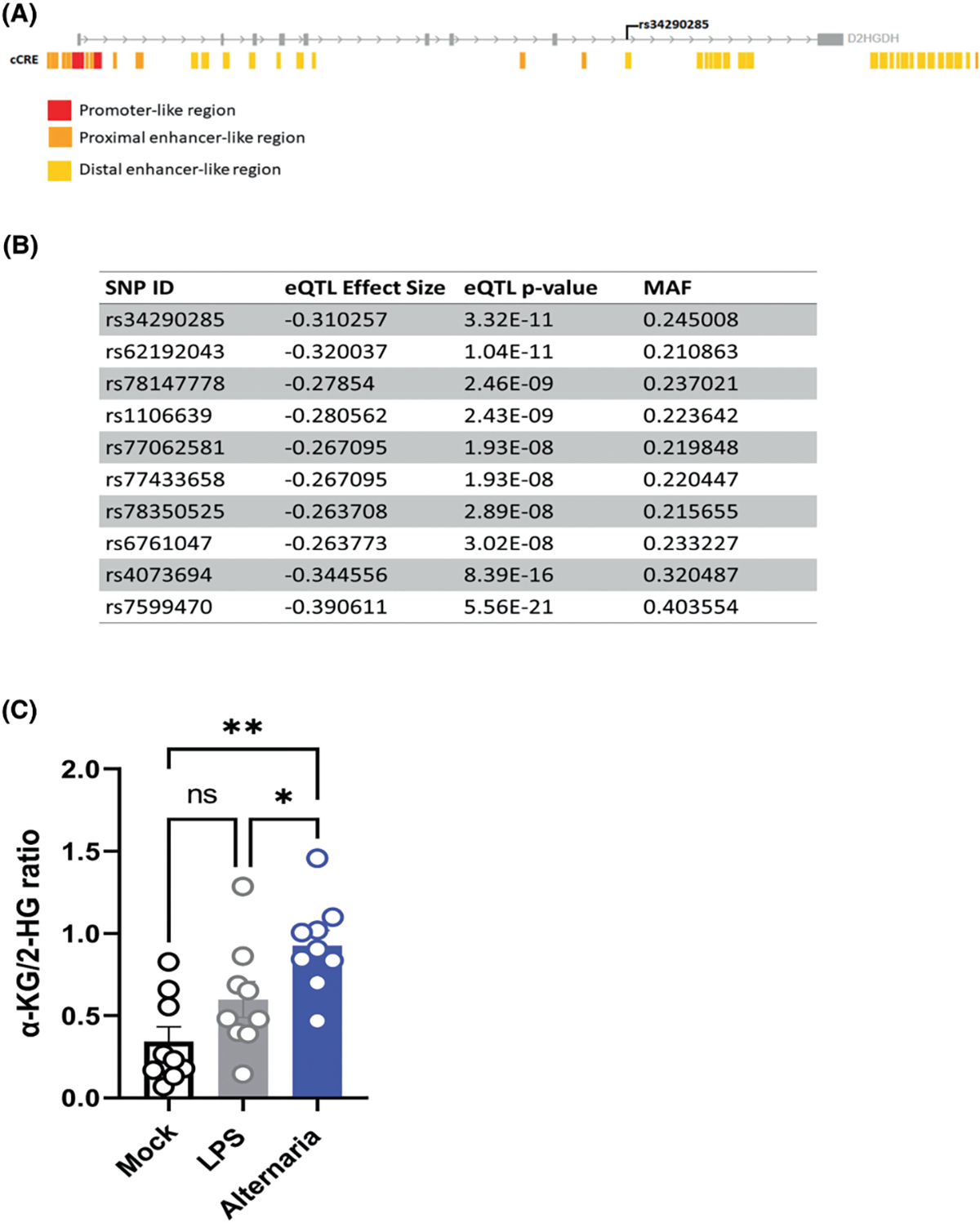
SNPs in the D2HGDH gene are associated with asthma. (A) Mapping of cCRE regions in the *D2HGDH* gene from ENCODE. Location of the sentinel SNP rs34290285 is noted. (B) Lung eQTL effect size, *p*-values, and minor allele frequencies for the 10 SNPs most strongly associated with asthma. (C) Untargeted metabolomics measuring the ratio of aKG/D-2HG in murine BMDCs following 16-h exposure to LPS or *Alternaria*.

To elucidate the functional consequences of these SNPs, the regulatory landscape of the *D2HGDH* locus was analyzed using the ENCODE database. We found that the sentinel SNP, rs34290285, resides within a candidate cis-regulatory element (cCRE) with distal enhancer-like features in human monocytes (Figure 1A). Previously described lung eQTL analysis of the 10 most significant SNPs revealed that these asthma risk alleles are associated with reduced expression of *D2HGDH* mRNA in the lung (Figure 1B).^3^ As the lead SNP, rs34290285 is also associated with *GAL3ST2*, the effects of *GAL3ST2* cannot be excluded.^3^ The downregulation of *D2HGDH* expression suggests that these SNPs may promote accumulation of D2HG, which is a potent endogenous inhibitor of α-KG-dependent dioxygenases, a group of histone demethylase and prolyl hydroxylase enzymes.^4^ Recent studies demonstrate that inhibition of these α-KG dependent histone demethylases in DCs results in a tolerogenic DC phenotype.^5^ D-2HG and α-KG, therefore, reciprocally regulate these dioxygenase enzymes to dynamically regulate gene expression via epigenetic mechanisms. Thus, we performed metabolomic analysis of murine bone marrow-derived dendritic cells following stimulation with LPS or the allergen *Alternaria* which revealed a significant increase in the ratio of α-KG to hydroxyglutarate following *Alternaria* treatment (Figure 1C).

**FIGURE 2:**
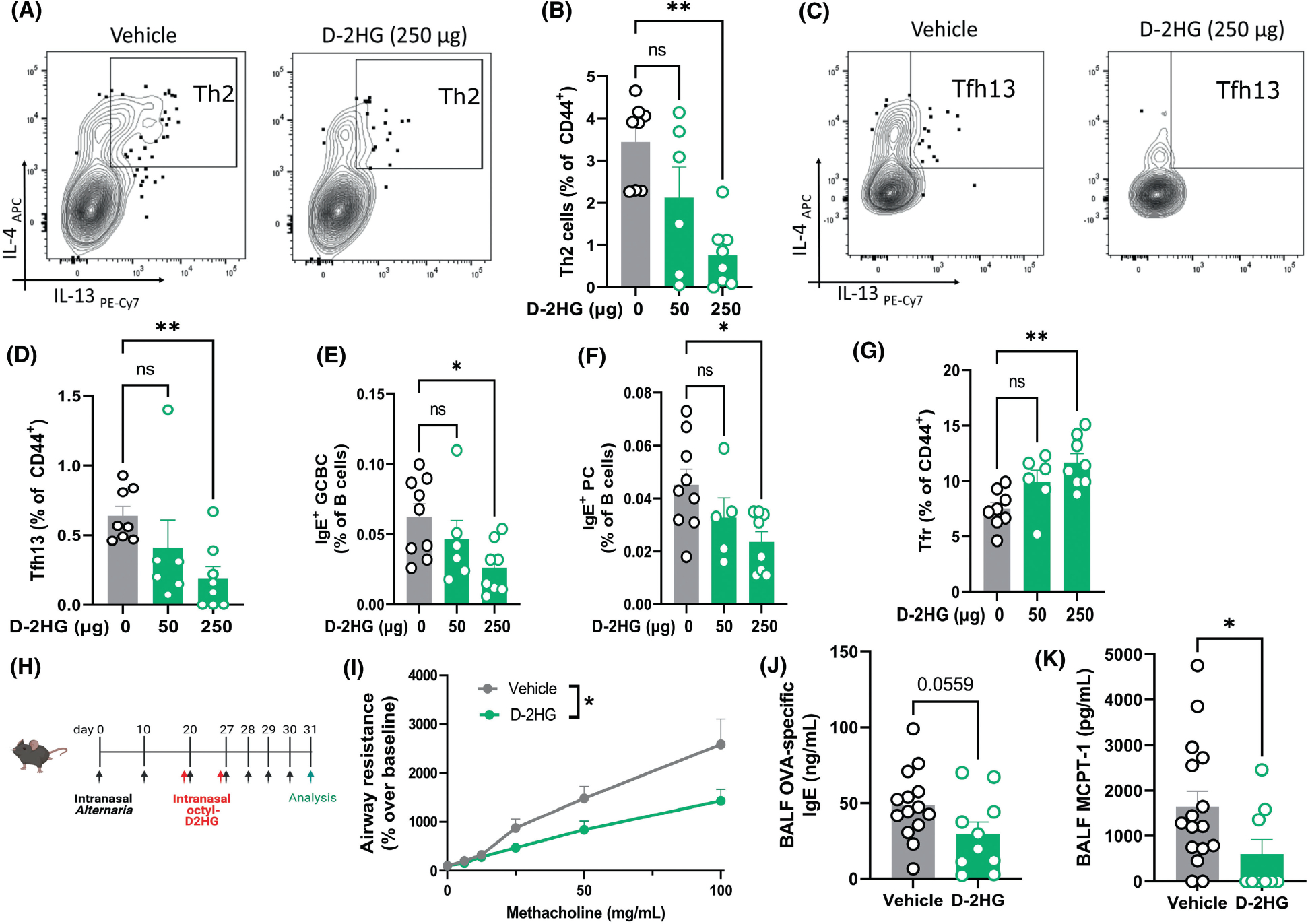
D-2HG reduces allergic sensitization to *Alternaria* in vivo. Mice were treated with octyl-D2HG prior to exposure to *Alternaria*. (A) Representative flow cytometry plots of Th2 cells (pre-gated on CD4^+^, TCRβ^+^, CD44^+^, B220^−^, CXCR5^−^) in the mLNs of mice 8 days after intranasal *Alternaria* sensitization. (B) Quantification of Th2 cells. (C) Representative flow cytometry plots of Tfh13 cells (pre-gated on CD4^+^, TCRβ^+^, CD44^+^, B220^−^, CXCR5^+^, PD-1^+^), (D) Tfh13 cells quantification. (E) IgE^+^ GCBCs (B220^+^, CD138^−^, CD95^+^, GL-7^+^, IgE^+^) and (F) IgE^+^ PCs (B220^+^, CD138^+^, IgE^+^), and (G) Tfr cells. (H) Schematic diagram of asthma model and mouse treatments. (I) Analysis of airway hyperreactivity on Day 31. (J) Bronchoalveolar lavage fluid (BALF) levels of antigen-specific IgE and (K) MCPT-1. *N* = 6–8 per group. *p < .05, **p < .01 by Kruskal–Wallis test and Dunn’s multiple comparisons test.

Therefore, we predicted that D-2HG may modulate DC function to alter allergic sensitization in a murine model of allergic airway inflammation. We found that intranasal administration of octyl-D-2HG, a cell permeable form of D-2HG, prior to intranasal exposure to *Alternaria* significantly reduced mediastinal lymph node (mLN) Th2, Tfh13, and Tfh2 cell polarization, but overall Tfh priming was unimpaired (Figure 2A–D. Figure S1A,B). Further, octyl-D2HG impaired mLN IgE^+^ germinal center B cell (GCBC) and IgE^+^ plasma cell (PC) differentiation (Figure 2E,F). IgG1^+^ GCBC and PC differentiation, however, was unaffected by octyl-D2HG administration (Figure S1C,D). Additionally, octyl-D-2HG administration promoted T follicular regulatory (Tfr) cell polarization, which act to restrain IgE production in allergic asthma (Figure 2G).^6^ Intranasal treatment of octyl-D2HG in a model of established murine allergic asthma resulted in a durable suppression of airway hyperreactivity for 4 days following treatment (Figure 2H,I) Additionally, mice treated with octyl-D2HG exhibited reduced levels of airway antigen-specific IgE and MCPT-1, indicating suppression of airway mast cell degranulation (Figure 2J,K).

Overall, these data demonstrate that D2HG critically regulates allergic sensitization and the polarization of Th2 and Tfh13 cells following allergen exposure. Further, we demonstrate that D-2HG induces Tfr cell differentiation in response to allergens in a murine model of allergic asthma, but it is not clear if this is the mechanism by which D2HG reduces allergic airway inflammation. Octyl-D2HG treatment in allergen-sensitized mice demonstrated significant attenuation of airway hyperreactivity and may therefore be a potential therapeutic approach for the treatment of allergic asthma and other IgE-mediated diseases.

## Supplementary Material

Supplemental methods and figures

## Data Availability

The data that support the findings of this study are available from the corresponding author upon reasonable request.

## References

[1] Kuruvilla ME, Lee FE, Lee GB. Understanding asthma phenotypes, Endotypes, and mechanisms of disease. Clin Rev Allergy Immunol. 2019;56:219–233. 30206782 10.1007/s12016-018-8712-1 PMC6411459

[2] Gowthaman U, Chen JS, Zhang B, Identification of a T follicular helper cell subset that drives anaphylactic IgE. Science. 2019;365:eaaw6433. 31371561 10.1126/science.aaw6433 PMC6901029

[3] Zhu Z, Lee PH, Chaffin MD, A genome-wide cross-trait analysis from UK biobank highlights the shared genetic architecture of asthma and allergic diseases. Nat Genet. 2018;50:857–864. 29785011 10.1038/s41588-018-0121-0 PMC5980765

[4] Du X, Hu H. The roles of 2-Hydroxyglutarate. Front Cell Dev Biol. 2021;9:651317. 33842477 10.3389/fcell.2021.651317 PMC8033037

[5] Donas C, Carrasco M, Fritz M, The histone demethylase inhibitor GSK-J4 limits inflammation through the induction of a tolerogenic phenotype on DCs. J Autoimmun. 2016;75:105–117. 27528513 10.1016/j.jaut.2016.07.011

[6] Clement RL, Daccache J, Mohammed MT, Follicular regulatory T cells control humoral and allergic immunity by restraining early B cell responses. Nat Immunol. 2019;20:1360–1371. 31477921 10.1038/s41590-019-0472-4 PMC6754271

