# Supplementary material for: D-2-hydroxyglutarate suppresses allergic sensitization in a murine model of experimental asthma
Source: Allergy. Author manuscript; Available in PMC 2023 Dec 20. (PMC10732592; doi:10.1111/all.15809)
Supplement: Supplemental methods and figures [file NIHMS1945919-supplement-Supplemental_methods_and_figures.docx]

**Materials and methods**

*Mice and airway exposure*

C57Bl/6J mice were purchased from the Jackson Laboratory. Mice were housed under specific-pathogen-free conditions in the Virginia Commonwealth University (VCU) barrier vivarium facility in accordance with the humane treatment of laboratory animals sets forth by the National Institutes of Health and the American Association for the Accreditation of Laboratory Animal Care. All mouse protocols were conducted with the permission and oversight of the VCU Institutional Animal Care and Use Committee. Mice were randomized for experiments and equal numbers of male and female mice were used unless otherwise specified. Experimental mice were 8–10 weeks old. Mice were anaesthetized with isoflurane and treated intranasally with the indicated dose of octyl-D2HG in a 50 µL volume one hour prior to intranasal administration of 10 µg *Alternaria*. Murine asthma models were induced via intranasal administration of 10 µg *Alternaria* (Greer Laboratories) + 100 µg OVA (Sigma) on day 0, followed by treatments of 5 µg *Alternaria* and 50 µg OVA on days 10, 20, and 27-30. Pulmonary function was testing by invasive airway monitoring on the Flexivent system (Scireq).

*SNP analysis*

cCre regions were identified in CD14^+^ monocytes in the ENCODE database based on high DNase-seq score, high H3K27ac, and low H3K4me3 score. The lead SNP, rs34290285 was mapped onto the *D2HGDH* locus. eQTLs were analyzed using the Broad Institute GTEx portal.

*Flow Cytometry*

Single cell suspensions were washed with PBS and live-dead staining was conducted using Zombie Aqua (Biolegend, 423102) according to manufacturer’s protocol. Cells were washed with FACs buffer (5% fetal bovine serum (FBS) in phosphate buffered saline (PBS) with 2mM EDTA). Fc receptors were blocked with 5μg 2.4G2 (130) for 10 minutes at 4°C. Antibodies were for 20 minutes at 4°C. Cells were washed two times with FACs buffer and fixed in Fixation Buffer (BioLegend, 420801) for 10 minutes at room temperature, or secondaries were added and incubated for 30 minutes at 4°C followed by fixation. For intracellular staining, following fixation, cells were permeabilized using Intracellular Stain Permeabilization Buffer (BioLegend, 421002) according to manufacturer’s protocol. Briefly, cells were stained for intracellular markers for 60 minutes at room temperature and washed two times with intracellular permeability buffer and then fixed using Fixation Buffer for 10 minutes at room temperature. Flow cytometry data was collected on an LSR Fortessa X-20 (BD Biosciences) and or FlowJo (BD Biosciences).

*T helper polarization analysis*

Mice were treated intranasally with D-2HG or vehicle control one hour prior to *Alternaria* exposure. Mice were euthanized 8 days later by isoflurane inhalation and mLN were collected and processed to generate single cell suspensions. Cells were stimulated with phorbol 12-myristate 13-acetate (PMA) (50 ng/ml) and ionomycin (1 μg/ml) in 96-well U bottom plates in complete IMDM media for 4 hours, and Brefeldin A was added for the last 3 hours. Cell suspensions were then processed for flow cytometric analysis as described above.

*Staining for IgE B cells*

Mice were treated intranasally with D-2HG or vehicle control one hour prior to *Alternaria* exposure. Mice were euthanized 8 days later and mLNs were collected and processed to generate single cell suspensions. Cell suspensions were incubated on ice for 10 minutes with viability dye and 2.4G2. Surface staining was performed with the addition of unlabeled anti-IgE (RME-1) to saturate Fc receptor bound IgE. Cells were then fixed with and permeabilized with BD intracellular staining kit and stained with fluorochrome conjugated anti-IgE (RME-1), anti-IgG1 overnight at 4°C.

*BMDC culture*

Hips, femurs, and tibiae were harvested from WT mice and crushed in a mortar and pestle. Cell suspensions were then filtered and plated at a density of 2-3x106 cells/mL in complete IMDM (IMDM medium with 10% FBS, 1.1 mg/mL β-Mercaptoethanol (Sigma-Aldrich), 2 mM L-glutamine (Thermo Fisher Scientific), and Antibiotic/Antimycotic (ThermoFisher)) in the presence of 200 ng/mL murine Flt3L (Peprotech). Cells were used for experiments after 10-12 days of culture.

*Untargeted metabolomics analysis*

BMDCs were stimulated with 10 µg/mL Alternaria or 100 ng/mL LPS for 16 hours, collected, washed, and snap frozen in liquid nitrogen. Snap-frozen cell pellets (2x106 cells/sample) were resuspended in 20% methanol/water and cells were lysed via sonication. Samples were centrifuged for 5 minutes at 5,000xg to pellet insoluble material. Supernatant was analyzed by liquid chromatography/tandem mass spectrometry using the ThermoFisher Q-Exactive HF system. Cell lysates and a pooled sample were resolved by liquid chromatography on a Vanquish UHPLC system on a silica column using a gradient from 50:50 acetonitrile:water with 0.1% formic acid to 1:99 acetonitrile: water with 0.1% formic acid at a flow rate of 300 microliters/min. Samples were analyzed in both positive and negative ion mode. In each ion mode, aliquots of each sample were pooled to generate representative MS2 spectra. Compound Discoverer v. 3.1. was used to deconvolute raw LC/MS data with respect to alignment and peak area determination. Compounds were identified relative to the MS2 spectra from the pooled sample. Statistical analysis was performed by Compound Discoverer v. 3.1.

*Statistical analysis*

The details of the replicates for each experiment are listed in the respective figure legends. The sample size in each experiment was determined based on the level of expected heterogeneity of the samples, the significance threshold (chosen at 0.05), the expected or observed difference, as well as previous publications. In all experiments, data are shown as mean ± SEM, and statistical analyses were performed using GraphPad Prism software (version 8.2.1). Unpaired Student’s t test, two-way ANOVA with Tukey’s multiple comparisons post-test, or Mann-Whitney test were used for statistical analysis as indicated in figure legends. One-way ANOVA with multiple comparisons test was used to compare the variance in more than two groups with one independent factor. Statistical significance is defined as ∗ p < 0.05, ∗∗ p < 0.01, ∗∗∗ p < 0.001, ∗∗∗∗ p < 0.0001, or NS (not significant).

**
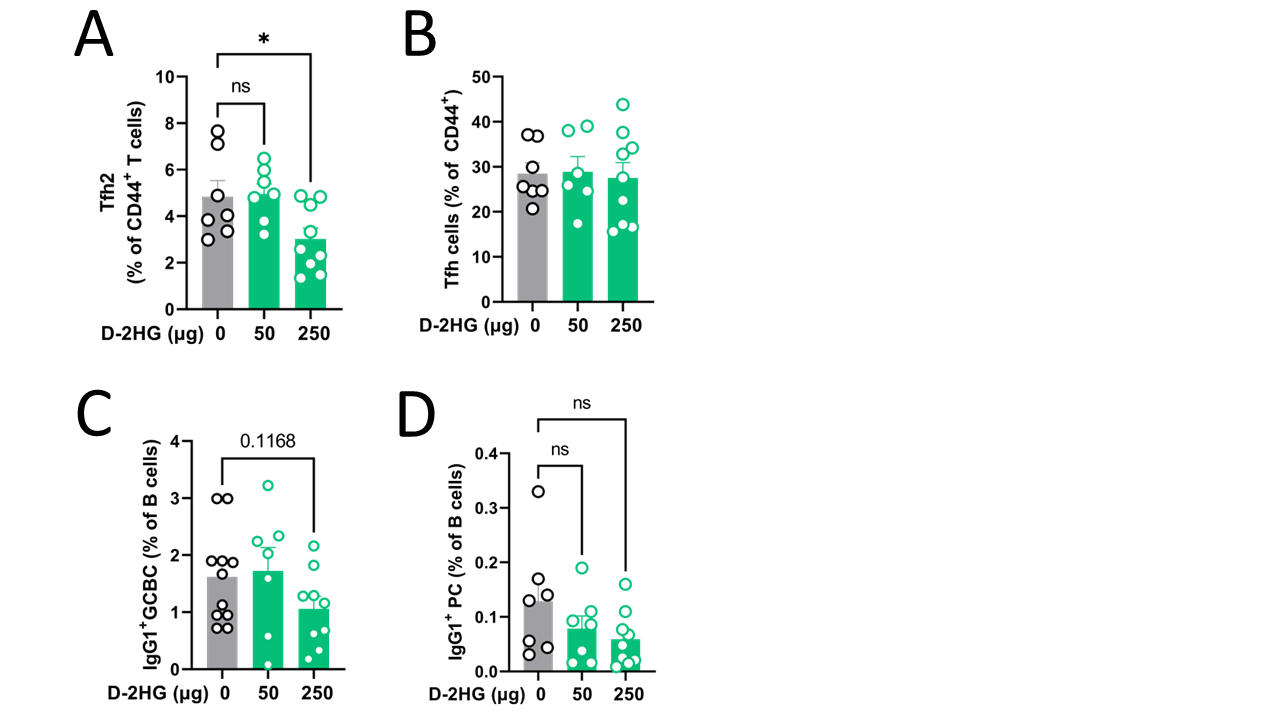
Figure S1. Octyl-D2HG does not suppress Tfh cell priming or IgG1 synthesis.**

Mice were treated with octyl-D2HG prior to exposure to *Alternaria.* **A** Quantification of Tfh2 cells (CD4^+^, TCRβ^+^, CD44^+^, B220^-^, CXCR5^+^, PD-1^+^, IL-4^+^, IL-13^-^) in the mLNs of mice 8 days after intranasal *Alternaria* sensitization. **B** Quantification of Tfh cells (CD4^+^, TCRβ^+^, CD44^+^, B220^-^, CXCR5^+^). **C** IgG1^+^ GCBCs (B220^+^, CD138^-^, CD95^+^, GL-7^+^, IgG1^+^) and **D** IgG1^+^ PCs (B220^+^, CD138^+^, IgG1^+^) in the mLNs. N=6-8 per group. *p<0.05, **p<0.01 by Kruskal-Wallis test and Dunn’s multiple comparisons test.
